# *Bacillus subtilis* natto NTU-18 attenuates atherosclerosis progression by modulating peripheral immune cell alterations

**DOI:** 10.1007/s00253-025-13604-0

**Published:** 2025-10-17

**Authors:** Jian-Da Lin, Yu-Zhen Ye, Sin-Ren Wang, Wen-Yi Kao, Kung-Ta Lee

**Affiliations:** 1 Department of Biochemical Science and Technology, National Taiwan University, Taipei, 10617 Taiwan; 2 Center for Computational and Systems Biology, National Taiwan University, Taipei, 10617 Taiwan; 3 Center for Advanced Computing and Imaging in Biomedicine, National Taiwan University, Taipei, 10617 Taiwan

**Keywords:** Atherosclerosis, Probiotics, *B. subtilis* natto NTU-18, High-dimensional flow cytometry, Effector memory T cells, Regulatory T cells (Tregs)

## Abstract

**Abstract:**

Atherosclerosis is a chronic inflammatory disease characterized by lipid accumulation and immune dysregulation, including expansion of pro-inflammatory monocytes and effector T cells, alongside reduced regulatory T cells (Tregs). *Bacillus subtilis* natto, a spore-forming probiotic, has shown anti-atherosclerotic effects, though its systemic immunomodulatory mechanisms remain unclear. In this study, we employed an AAV-mPCSK9-induced murine atherosclerotic model to investigate the effects of daily *B. subtilis* natto NTU-18 administration over 16 weeks. High-dimensional flow cytometry using two 13-marker panels enabled longitudinal profiling of 18 peripheral immune cell subsets across lymphoid and myeloid compartments. While no significant changes in serum cholesterol and mild decrease of body weight were observed, *B. subtilis* natto NTU-18-treated mice presented a significant reduction in aortic lesion area compared to PBS-treated controls. Immune profiling revealed a transient expansion of peripheral myeloid cells and CD44⁺ trained CD8⁺ T cells, followed by increased frequencies of naïve CD8⁺ T cells and reduced central/effector memory subsets at longer time point treatment. In the CD4⁺ T cell compartment, a transient increase in trained cells was accompanied by a sustained enrichment of CD25⁺CD4⁺ Tregs throughout the daily *B. subtilis* natto NTU-18 treatment. In contrast, no significant differences were observed in Ly6C⁻ or Ly6C⁺ monocytes, neutrophils, or eosinophils. These findings suggest that *B. subtilis* natto NTU-18 attenuates atherosclerosis progression not through lipid lowering or broad myeloid modulation, but via targeted reprogramming of peripheral T cell responses. This work provides mechanistic insight into the immunotherapeutic potential of *B. subtilis* natto NTU-18 in atherosclerosis prevention and treatment.

**Key points:**

*• B. subtilis natto NTU-18 significantly reduces aortic plaque burden in atherosclerotic mice without affecting serum cholesterol levels*.

*• B. subtilis natto NTU-18 induces transient immune remodeling, marked by early expansion of trained CD8⁺ and CD4⁺ T cells, followed by increased naïve and regulatory T cells*.

*• The atheroprotective effect is primarily mediated through adaptive immunity as myeloid subsets remain unchanged throughout B. subtilis natto NTU-18 treatment*.

## Introduction

Atherosclerosis is a chronic inflammatory disease induced by lipid accumulation and immune cell infiltration, ultimately leading to aortic plaque formation and vascular dysfunction (Ajoolabady et al. 2024). Systemic immune dysregulation, particularly the expansion of pro-inflammatory CD14⁺CD16⁺ monocytes (analogous to Ly6C^hi^ monocytes in mice) and effector memory CD4⁺/CD8⁺ T cells, alongside a reduction in regulatory T cells (Tregs), has been strongly associated with plaque progression and instability (Ammirati et al. 2012; Foks et al. 2015; Hilgendorf et al. 2015). Recent advances in high-dimensional flow cytometry have enabled comprehensive profiling of peripheral immune landscapes, revealing dynamic shifts in both innate and adaptive immune compartments throughout disease progression. These peripheral immune signatures provide insight into atherosclerotic pathogenesis and may serve as biomarkers for monitoring therapeutic efficacy.

*Bacillus subtilis* natto, a spore-forming probiotic derived from the traditional Japanese food natto, has shown promising anti-atherosclerotic effects through multiple mechanisms in both preclinical and clinical studies. In LDL (low-density lipoprotein) receptor-deficient mice reconstituted with iRFP (infrared fluorescent protein) -labeled hematopoietic cells, administration of high vitamin K₂-producing *B. subtilis* natto strains significantly reduced plaque formation (Kawamata et al. 2023). Consistently, a clinical study involving 1062 participants demonstrated that daily supplementation with nattokinase (NK) or NK-enriched natto effectively lowered lipid levels and attenuated atherosclerosis in individuals with pre-existing plaques (Chen et al. 2022). These effects have been linked to enhanced thrombolytic activity, antioxidant and anti-apoptotic properties, reduced intestinal inflammation, and inhibition of LDL oxidation. In vitro studies further support the immunomodulatory potential of *B. subtilis* natto, showing that B4 spores enhance nitric oxide and cytokine production in murine macrophages (RAW264.7), while both live and UV-inactivated strains promote anti-inflammatory cytokine expression in colorectal epithelial cell line model (Caco-2) (Abedi Elkhichi et al. 2024; Xu et al. 2012).

To investigate how *B. subtilis* natto NTU-18 modulates systemic immune responses relevant to atherosclerosis, we employed high-dimensional flow cytometry using two 13-marker panels to profile peripheral immune cells from *B. subtilis* natto NTU-18-treated atherosclerotic mice. This approach enabled the delineation of over 15 immune cell subsets and their activation states. Our study aims to elucidate the anti-atherosclerotic mechanisms of *B. subtilis* natto NTU-18 through its impact on systemic immune modulation, thereby providing a theoretical basis for its application in the prevention and treatment of atherosclerosis and lipid metabolism disorders.

## Materials and methods

### *B. subtilis* natto NTU-18 powder preparation

*B. subtilis* natto (BCRC 80390) fermentative powder was provided by Taiwan Microzyme Co., LTD. (Taipei City, Taiwan) according to high extracellular vehicles (EVs) fermentation procedure (EVs > 1 × 10^12^ particles/g). The fermentation process was modified from our previous study (Kuo et al. 2006, 2012). Briefly, the black soybeans were milled using a grinder to produce black soybean powder for black soymilk preparation. The *B. subtilis* natto NTU-18 strain (BCRC 80390, Bioresource Collection and Research Center, Taiwan) was maintained on nutrient broth (NB) slants at 4 °C. Seed cultures were prepared by incubating *B. subtilis* natto NTU-18 in 100 ml NB at 37 °C, 125 rpm for 12 h, then used to inoculate a 7-l fermenter with 5 l of 5% (w/v) black soymilk, fermented at 37 °C with 1.0 v.v.m. aeration and 800 rpm agitation. Whole fermentation products were spray-dried into powder before being provided to atherosclerotic mice.

### Mouse model

 Specific pathogen-free (SPF) male C57BL/6 mice (8 weeks old; National Center for Biomodels, Taipei, Taiwan) were used in this study. The animals were housed at 22 ± 2 °C under a 12-h light/12-h dark cycle with free access to food and water. This study was approved by the Institutional Animal Care and Use Committee of Fu Jen Laboratory Animal Centre (Taipei, Taiwan) (IACUC permission no. P11004). Murine atherosclerosis progression was induced by a novel model that bypasses the need for germline-deficient strains such as LDL receptor–deficient (*Ldlr⁻/⁻*) or apolipoprotein E-deficient (*Apoe⁻/⁻*) mice (Bjorklund et al. 2014; Roche-Molina et al. 2015). This approach leverages the activity of proprotein convertase subtilisin/kexin type 9 (PCSK9), which promotes hepatic LDL receptor degradation (Li et al. 2007). A single injection of recombinant adeno-associated virus encoding a gain-of-function PCSK9 mutant, either human PCSK9D374Y or mouse PCSK9D377Y (AAVmPCSK9), effectively reduces LDL receptor expression, elevates plasma LDL cholesterol, and induces atherosclerosis in mice (Bjorklund et al. 2014; Lin et al. 2019; Peled et al. 2017; Roche-Molina et al. 2015). We used a single intraperitoneal injection of AAV-mPCSK9 (ssAAV8/hAAT-mPCSK9D377Y; AAV core facility, Institute of Biomedical Sciences, Academia Sinica, Taipei, Taiwan) at a dose of 5 × 10^11^ viral particles per mouse. The plasmid used for viral production was obtained from Addgene (Plasmid #58,376; pAAV/D377Y-mPCSK9, Watertown, MA, USA) and generously provided by Dr. Mi-Hua Tao. Following injection, mice were fed a Western diet (Research Diets, Cat# D12079Bi, New Brunswick, NJ, USA) for 18 weeks. Beginning at week 2, mice were orally gavaged daily with either PBS (*n* = 7) or *B. subtilis* natto NTU-18 (BCRC 80390, 0.1 g/kg; *n* = 4) (Kuo et al. 2006; Kuo and Lee 2008) for 16 weeks then mice were sacrificed on 18 weeks post AAV-mPCSK9 and Western diet. Body weight was monitored weekly throughout the feeding period.

### Cholesterol measurement

Submandibular blood was collected from atherosclerotic mice fed a Western diet for 0, 2, 6, 12, and 18 weeks. Heparin was added immediately after collection to prevent coagulation. Plasma was obtained by centrifuging the blood samples at 1500 rpm for 10 min at 4 °C. Cholesterol levels were measured using a colorimetric assay (FUJIFILM Wako, Cat# 639–50,981, Chūō-ku, Osaka, Japan). To prepare the samples, 2 µl of plasma was mixed with 8 µl of distilled water to achieve a fivefold dilution. Then, 200 µl of Color Reagent Solution (FUJIFILM Wako, Cat# 639–50,981, Chūō-ku, Osaka, Japan) was added to each sample, followed by incubation at 37 °C for 5 min. Absorbance was measured at 600 nm using a microplate reader. Cholesterol concentrations were determined by comparing absorbance values to a standard curve. Statistical significance between groups at each time point was assessed using the unpaired Mann–Whitney *U* test (*p < 0.05).

### Histological analysis

The aortic roots were harvested from mice and embedded in OCT compound (Leica Biosystems, Cat# 14,020,108,926, Deer Park, IL, USA). Samples were stored at − 20 °C until sectioning. Frozen tissue Sects. (6-µm thickness) were fixed in acetone for 10 min, rinsed briefly in 60% isopropanol, and stained with hematoxylin for 30–90 s to visualize nuclear morphology. Excess stain was removed by a quick rinse in water, followed by differentiation in acid alcohol and bluing in ammonia water to achieve crisp nuclear detail. Sections were then counterstained with eosin for 10–45 s to highlight cytoplasmic and extracellular structures. After counterstaining, tissues were rapidly dehydrated through graded ethanols, cleared in xylene, and mounted with a resinous medium. Intimal lesions and stained areas were visualized using an inverted fluorescence microscope (OLYMPUS CKX53, Hachioji-shi, Tokyo Japan) and quantified using ImageJ software (NIH, Bethesda, MD, USA).

### Blood cell isolation and flow cytometry

 Submandibular blood was collected from atherosclerotic mice, and heparin was added immediately after collection to prevent coagulation. Plasma was separated by centrifuging the blood samples at 1500 rpm for 10 min at 4 °C. The cell pellet was resuspended in 2 ml of RBC (red blood cell) lysis buffer to lyse red blood cells. After incubation at room temperature for 5 min, 10 ml of PBS (phosphate-buffered saline) was added to stop the lysis. Cells were then centrifuged at 1500 rpm for 5 min at 4 °C, and the pellet was resuspended in FACS (fluorescence-activated cell sorting) buffer containing 1 × HBSS (Hank’s balanced salt solution), 1% BSA (bovine serum albumin), 1 mM EDTA (ethylenediaminetetraacetic acid), 20 mM HEPES (4-(2-hydroxyethyl)−1-piperazineethanesulfonic acid), and 1 mM sodium pyruvate. The single-cell suspension was filtered through a 35 µm cell strainer to ensure removal of debris and cell aggregates, yielding a preparation suitable for downstream flow cytometry analysis. Cells were subsequently stained with lymphoid and myeloid antibody panels and analyzed by flow cytometry.

**Lymphoid panel:** The following antibodies are from BioLegend (San Diego, CA, USA): BV421-conjugated anti-mouse CX3CR1 antibody (Clone: CXCR3-173, Cat. 126,529), BV510-conjugated anti-mouse CD27 antibody (Clone: LG.3A10, Cat. 124,229), BV605-conjugated anti-mouse KLRG1 antibody (Clone: 2F1, Cat. 138,419), BV650-conjugated anti-mouse CD8 antibody (Clone: 53–6.7, Cat. 100,742), PerCP/Cy5.5-conjugated anti-mouse CD127 antibody (Clone: A7R34, Cat. 135,022), Alexa Fluor 488-conjugated anti-mouse CD45 antibody (Clone: S18009D, Cat. 160,306), Phycoerythrin (PE)-conjugated anti-mouse CD44 antibody (Clone: IM7, Cat. 103,008), PE-Cy5-conjugated anti-mouse CD3 antibody (Clone: 17A2, Cat. 100,274), PE/Dazzle 594 conjugated anti-mouse CD19 antibody (Clone: 6D5, Cat. 115,554), Allophycocyanin (APC)-conjugated anti-mouse CD62L antibody (Clone: MEL-14, Cat. 104,412), APC-Cy7-conjugated anti-mouse CD4 antibody (Clone: GK1.5, Cat. 100,414), Alexa Fluor 700-conjugated anti-mouse CD69 antibody (Clone: H1.2F3, Cat. 104,539). PE/Cy7-conjugated anti-mouse CD25 antibody (Clone: PC61.5, eBioscience (San Diego, CA, USA), Cat. 25–0251-82) and Live/Dead Blue viability dye (Thermo Fisher (Waltham, MA, USA), Cat. L34962). **Myeloid panel:** The following antibodies are from BioLegend (San Diego, CA, USA): BV510-conjugated anti-mouse B220 antibody (Clone: RA3-6B2, Cat. 103,248), BV605-conjugated anti-mouse TCR-b antibody (Clone: H57-597, Cat. 109,241), BV650-conjugated anti-mouse CD11c antibody (Clone: N418, Cat. 117,339), PerCP/Cy5.5-conjugated anti-mouse CD301 antibody (Clone: LOM-14, Cat. 145,710), Alexa Fluor 488-conjugated anti-mouse Ly6G antibody (Clone: 1A8, Cat. 127,626), PE-conjugated anti-mouse CX3CR1 antibody (Clone: SA011F11, Cat. 149,006), PE-Cy5-conjugated anti-mouse CD11b antibody (Clone: M1/70, Cat. 101,210), PE/Dazzle 594 conjugated anti-mouse CD45 antibody (Clone: 30-F11, Cat. 103,146), APC-conjugated anti-mouse PDL2 antibody (Clone: TY25, Cat. 107,210), APC-Cy7-conjugated anti-mouse MHCII antibody (Clone: M5/114.15.2, Cat. 107,628), Alexa Fluor 700-conjugated anti-mouse F4/80 antibody (Clone: BM8, Cat. 123,130). BV421-conjugated anti-mouse Siglec-F antibody (Clone: E50-2440, BD BioScience (Franklin Lakes, NJ, USA), Cat. 562,681), PE/Cy7-conjugated anti-mouse Ly6C antibody (Clone: AL21, BD BioScience (Franklin Lakes, NJ, USA), Cat. 560,593), and Live/Dead Blue viability dye (Thermo Fisher (Waltham, MA, USA), Cat. L34962).

## Results

### Effects of daily *B. subtilis* natto NTU-18 administration on body weight in AAVmPCSK9-induced atherosclerotic mice

**Fig. 1:**
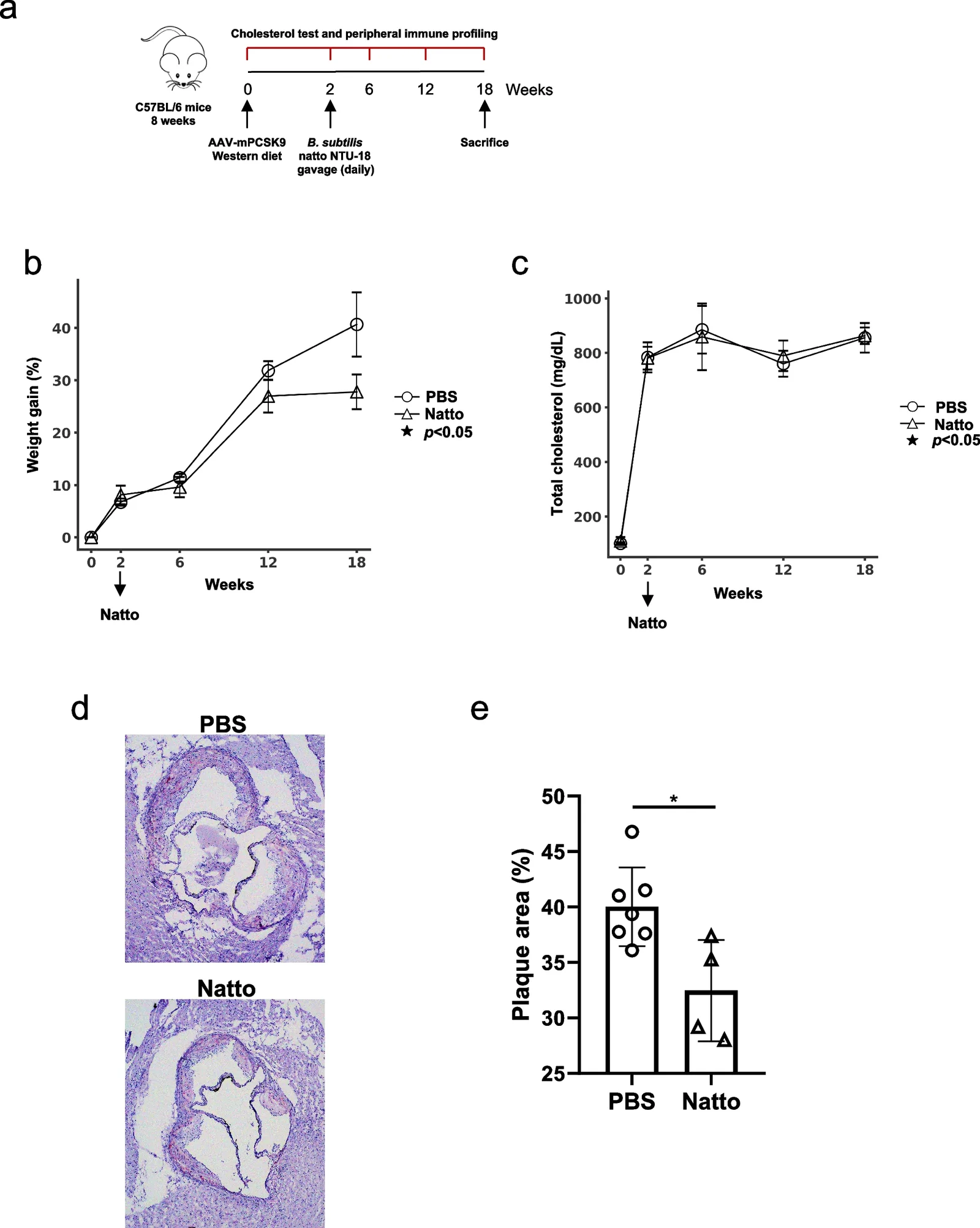
Oral administration of *B. subtilis* natto NTU-18 attenuates aortic plaque progression in the murine atherosclerosis model. **a** Experimental timeline. Wild-type C57BL/6J mice were injected with AAV-mPCSK9 and fed a Western diet (WD) from week 0. Daily oral gavage with *B. subtilis* natto NTU-18 or PBS was started at week 2 and continued daily throughout the study. Blood was collected at indicated time points for cholesterol quantification and immune profiling, then mice were sacrificed at week 18. **b** Body weight gain over time in PBS- and *B. subtilis* natto NTU-18-treated mice. **c** Total plasma cholesterol levels measured at 0, 2, 6, 12, and 18 weeks. **d** Representative hematoxylin and eosin (H&E)-stained cross-sections of aortic root lesions from PBS- and *B. subtilis* natto NTU-18-treated mice at week 18. **e** Quantification of aortic plaque area expressed as percentage of total aortic root area. Data are presented as mean ± SEM. Each symbol represents an individual mouse (*n* = 7, PBS-treated group; *n* = 4, *B. subtilis* natto NTU-18-treated group). Statistical analysis was performed using unpaired two-tailed Student’s *t*-test; *p < 0.05

The experimental scheme for atherosclerosis induction and daily *B. subtilis* natto NTU-18 treatments started at week 2 is showed as Fig. 1a. After 18 weeks of Western diet feeding and 16 weeks of daily *B. subtilis* natto NTU-18 treatments, AAVmPCSK9-injected C57BL/6 mice developed significant weight gain, consistent with the establishment of diet-induced atherosclerosis (Fig. 1b). Mice treated with *B. subtilis* natto NTU-18 showed a trend toward reduced body weight gain compared to PBS-treated controls. However, the difference did not reach statistical significance (Fig. 1b).

### Effect of daily *B. subtilis* natto NTU-18 administration on serum cholesterol levels

Hypercholesterolemia is a key driver of atherosclerosis development. To assess whether *B. subtilis* natto NTU-18 influences systemic lipid metabolism, serum cholesterol levels were measured throughout several time points. Daily administration of *B. subtilis* natto NTU-18 did not result in significant alterations in total cholesterol when compared to PBS-treated controls (Fig. 1c).

### Daily *B. subtilis* natto NTU-18 administration reduces atherosclerotic lesion formation

The anti-atherogenic effect of *B. subtilis* natto NTU-18 was evaluated by quantifying atherosclerotic lesion formation in the aortic root. Mice receiving *B. subtilis* natto NTU-18 exhibited a significant reduction in lipid-rich lesion area compared to PBS-treated controls (Fig. 1d and e). These findings indicate that *B. subtilis* natto NTU-18 confers a protective effect against atherosclerosis progression.

### High-dimensional flow cytometry reveals immune landscape monitoring following B. subtilisnatto NTU-18 administration

**Fig. 2:**
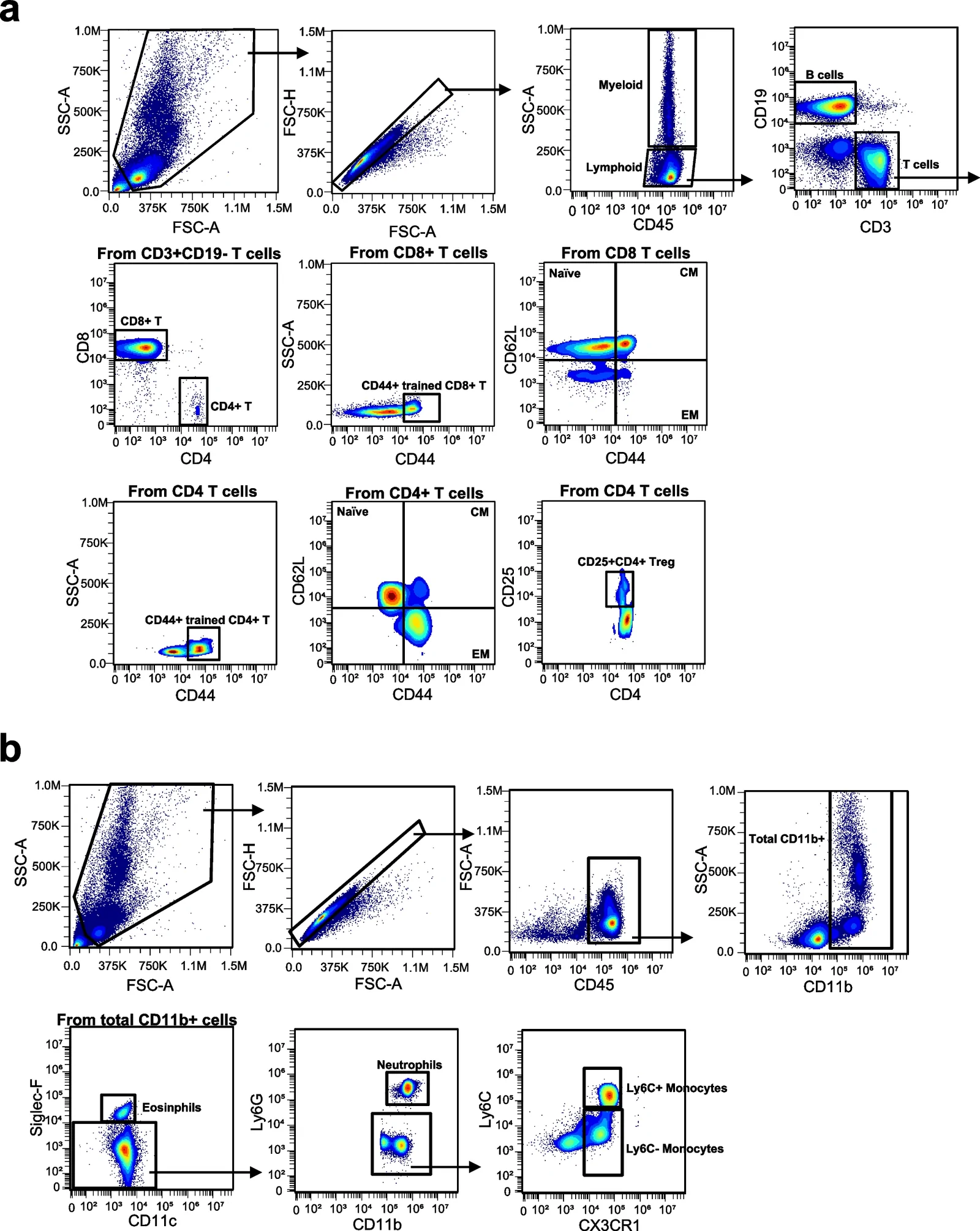
Gating strategies for peripheral immune cell profiling by high-parameter flow cytometry. **a** Lymphocyte gating and subpopulation identification. Peripheral blood mononuclear cells were first gated based on forward and side scatter (FSC-A vs. SSC-A), followed by singlet discrimination (FSC-A vs. FSC-H). CD45⁺ leukocytes were divided into lymphoid and myeloid populations. CD3⁺CD19⁻ T cells were further subdivided into CD4⁺ and CD8⁺ subsets. Effector memory (EM), central memory (CM), and naïve CD8⁺ and CD4⁺ T cells were identified based on CD44 and CD62L expression. Regulatory T cells (Tregs) were defined as CD25⁺CD4⁺ within the CD4⁺ T cell compartment. **b** Myeloid cell gating. After singlet and CD45⁺ gating, CD11b⁺ myeloid cells were analyzed. Eosinophils were defined as CD11c^−^Siglec-F⁺, neutrophils as Ly6G⁺CD11b⁺, and monocyte subsets as Ly6C⁺CX3CR1^+^ (Ly6C⁺ monocytes) and Ly6C⁻CX3CR1⁺ (Ly6C⁻ monocytes)

Alterations in peripheral immune cell populations have been implicated in atherosclerosis progression. To evaluate the immunomodulatory effects of daily *B. subtilis* natto NTU-18 administration, peripheral blood was collected at serial time points (weeks 0, 2, 6, 12, and 18) from both control and treatment groups. High-dimensional flow cytometry was employed to comprehensively profile the circulating immune landscape. This approach enabled the simultaneous identification and quantification of 18 distinct lymphoid and myeloid cell populations, including total T cells, B cells, and myeloid cells, as well as detailed subsets of CD4⁺ and CD8⁺ T cells. These subsets comprised naïve (CD62L⁺CD44⁻), central memory (CD62L⁺CD44⁺), effector memory (CD62L⁻CD44⁺), and trained (CD44⁺) phenotypes (Fig. 2a). Additional populations analyzed included CD25⁺CD4⁺ regulatory T cells (Tregs), eosinophils, neutrophils, and both Ly6C⁻ and Ly6C⁺ monocytes (Fig. 2b). These analyses enable the screening of the dynamics of peripheral immune subsets during *B. subtilis* natto NTU-18 treatment in atherosclerotic mice (Fig. 2). Despite the smaller number of mice in the *B. subtilis* natto NTU-18 group (*n* = 4) and PBS-treated control group (*n* = 7), longitudinal high-dimensional immune phenotyping, including multiple cell types, yielded highly consistent results across individuals, reflected by smaller standard errors of the mean (SEM). The repeated detection of comparable immune dynamics at multiple time points (weeks 0, 2, 6, 12, and 18) further underscores the robustness of these findings.

### Effects of *B. subtilis* natto NTU-18 administration on peripheral lymphoid and myeloid cell dynamics in atherosclerotic mice

**Fig. 3:**
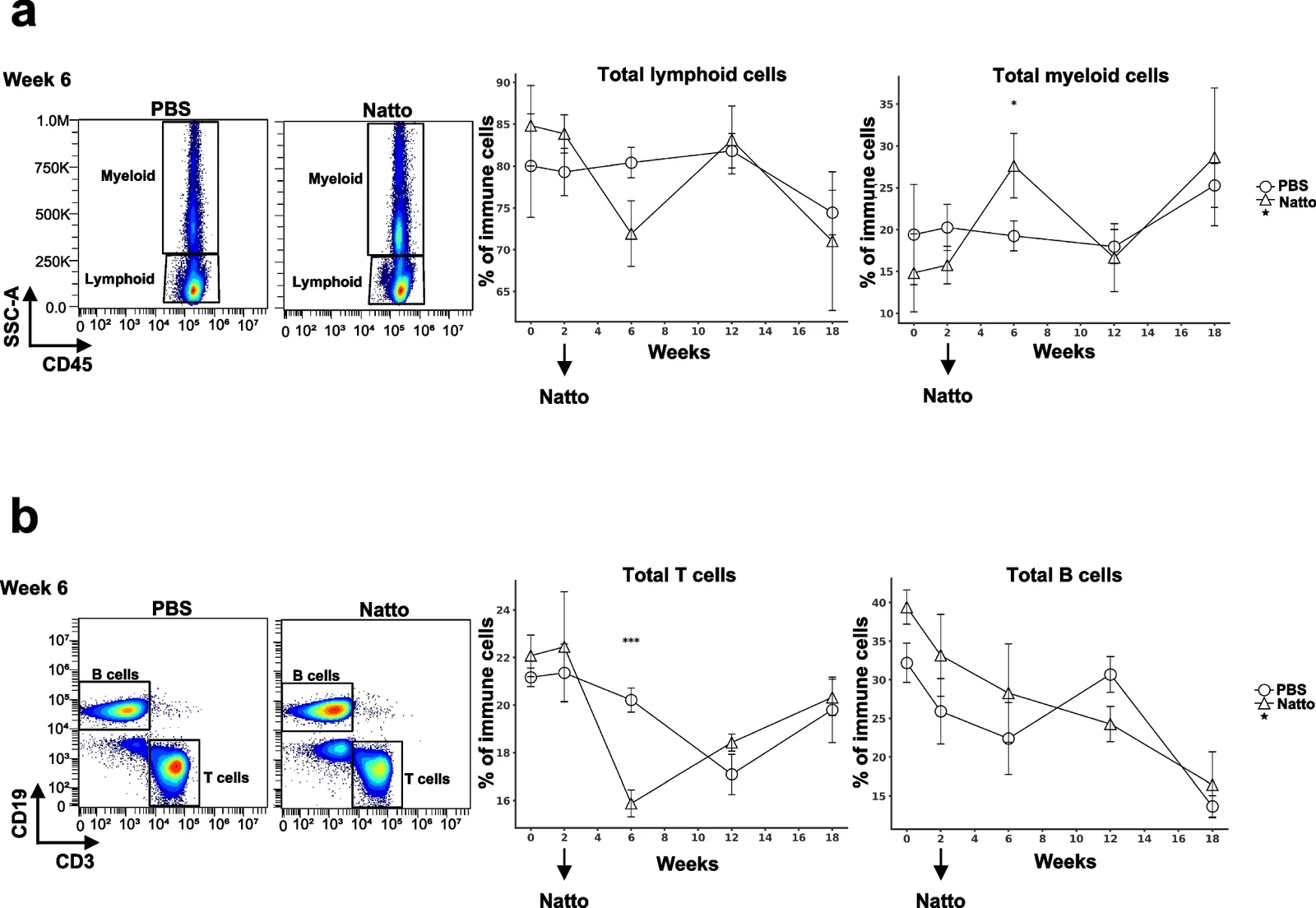
*B. subtilis* natto NTU-18 treatment modulates peripheral lymphoid and myeloid cell composition during atherosclerosis progression. **a** Representative density plots at week 6 and longitudinal quantification of total lymphoid and myeloid cells in peripheral blood at indicated time points. CD45⁺ cells were gated into lymphoid and myeloid populations based on side scatter (SSC-A) and CD45 expression. **b** Representative density plots at week 6 and longitudinal quantification of CD3⁺ T cells and CD19⁺ B cells from lymphoid populations at indicated time points. Data represent mean ± SEM (*n* = 7, PBS-treated group; *n* = 4, *B. subtilis* natto NTU-18-treated group). Statistical analysis was performed using unpaired two-tailed Student’s *t*-test at each time point; *p < 0.05, ***p < 0.001

High-dimensional flow cytometry analysis based on myeloid and lymphoid lineage gating revealed a transient expansion of total peripheral myeloid cells following 4 weeks of daily *B. subtilis* natto NTU-18 administration (Fig. 3a). In contrast, a significant reduction in total lymphoid cells was observed during the same period, primarily driven by a decrease in circulating T cell populations (Fig. 3b). Notably, by week 6 and beyond, no significant differences in total myeloid or lymphoid cell frequencies were detected between *B. subtilis* natto NTU-18-treated and PBS control groups. These findings suggest that *B. subtilis* natto NTU-18 induces a transient modulation of peripheral immune composition during the early phase of treatment, which may contribute to establishing a more regulated immune environment. The normalization of immune cell profiles over time implies the induction of a homeostatic immune response, potentially contributing to the observed attenuation of atherosclerosis progression by week 18.

### Effects of *B. subtilis* natto NTU-18 administration on dynamics of peripheral trained CD8^+^ T cell subsets in atherosclerotic mice

**Fig. 4:**
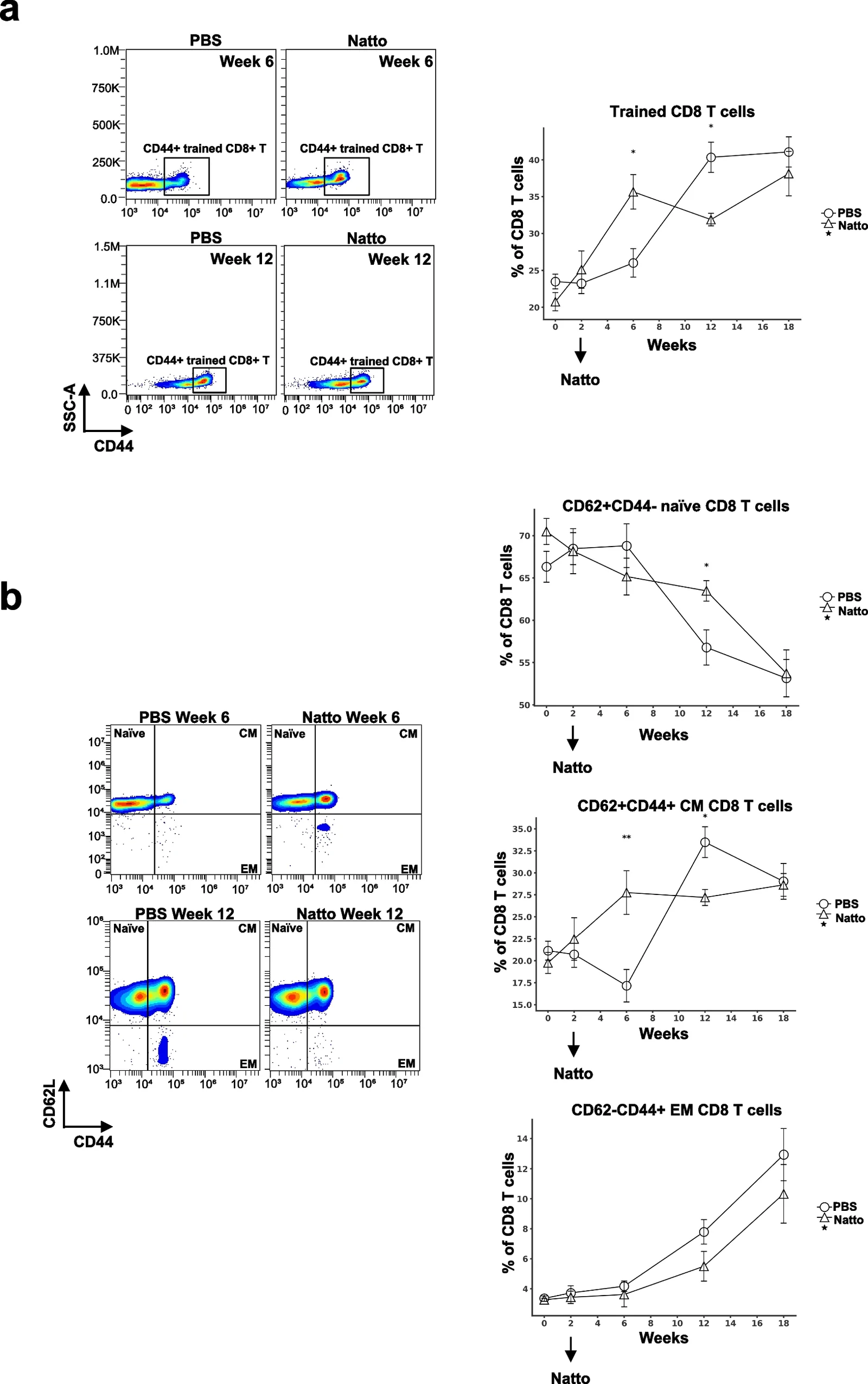
*B. subtilis* natto NTU-18 modulates trained and memory CD8⁺ T cell dynamics during atherosclerosis progression. **a** Representative density plots and longitudinal quantification of CD44⁺ “trained” CD8⁺ T cells in peripheral blood at indicated time point. **b** Representative density plots and longitudinal frequency analysis of CD8⁺ T cell subsets defined by CD44 and CD62L expression at indicated time point: naïve (CD62L⁺CD44⁻), central memory (CM; CD62L⁺CD44⁺), and effector memory (EM; CD62L⁻CD44⁺). Data represent mean ± SEM (*n* = 7, PBS-treated group; *n* = 4, *B. subtilis* natto NTU-18-treated group). Statistical analysis was performed using unpaired two-tailed Student’s *t*-test at each time point; *p < 0.05, **p < 0.01

Systemic immune dysregulation, characterized by the expansion of effector memory CD4⁺ and CD8⁺ T cells and a concomitant reduction in regulatory T cells (Tregs), has been strongly implicated in promoting plaque progression and instability (Ammirati et al. 2012; Foks et al. 2015). Peripheral CD8⁺ T cell populations were profiled in *B. subtilis* natto NTU-18-treated mice and compared to PBS-treated atherosclerotic controls. A transient expansion of CD44⁺ trained CD8⁺ T cells was observed following 4 weeks of *B. subtilis* natto NTU-18 administration, which was subsequently followed by a significant reduction in circulating trained CD8⁺ T cells after 10 weeks of treatment compared to PBS controls (Fig. 4a). Similar dynamic changes were observed within the central memory CD8⁺ T cell compartment, with *B. subtilis* natto NTU-18-treated mice presenting a higher proportion of naïve CD8⁺ T cells and a corresponding decrease in effector memory CD8⁺ T cells at week 10 compared to controls (Fig. 4b). These findings suggest that daily *B. subtilis* natto NTU-18 administration for 10 weeks promotes the rebalancing of peripheral CD8⁺ T cell subsets toward a less activated, more naïve phenotype, which contribute to the attenuation of atherosclerosis progression.

### Effects of *B. subtilis* natto NTU-18 administration on dynamics of peripheral trained and regulatory CD4⁺ T cell subsets in atherosclerotic mice

**Fig. 5:**
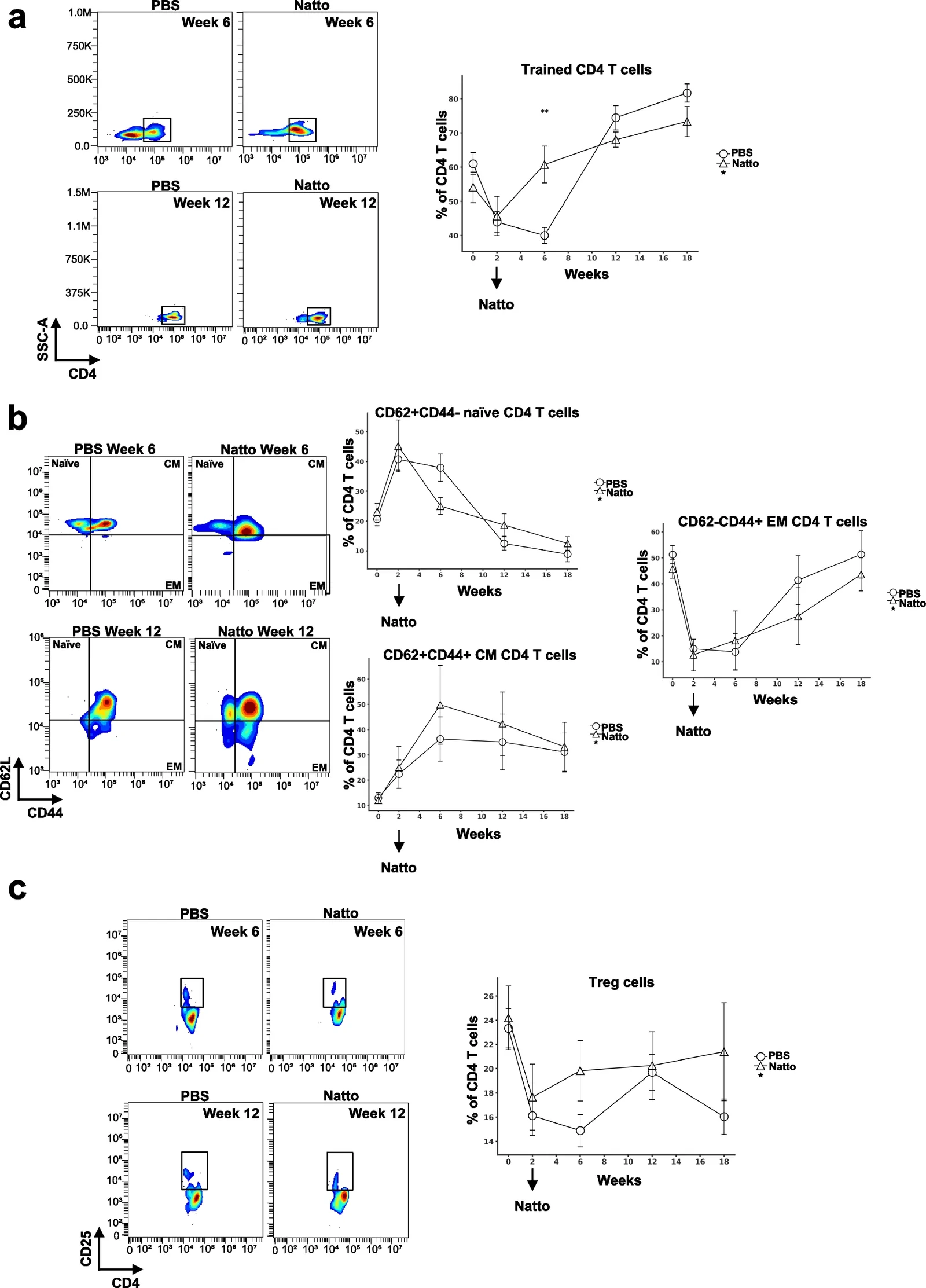
*B. subtilis* natto NTU-18 modulates trained and regulatory CD4⁺ T cell dynamics during atherosclerosis progression. **a** Representative density plots and longitudinal quantification of CD44⁺ “trained” CD4⁺ T cells in peripheral blood at indicated time point. **b** Representative density plots and longitudinal frequency analysis of CD4⁺ T cell subsets defined by CD44 and CD62L expression at indicated time point: naïve (CD62L⁺CD44⁻), central memory (CM; CD62L⁺CD44⁺), and effector memory (EM; CD62L⁻CD44⁺). **c** Representative density plots and longitudinal quantification of CD25⁺ CD4⁺ regulatory T cells (Tregs) in peripheral blood at indicated time point. Data are presented as mean ± SEM (*n* = 7, PBS-treated group; *n* = 4, *B. subtilis* natto NTU-18-treated group). Statistical analysis was performed using unpaired two-tailed Student’s *t*-test at each time point; **p < 0.01

Next, we analyzed the peripheral CD4⁺ T cell subsets in *B. subtilis* natto NTU-18-treated mice and compared them to PBS-treated atherosclerotic controls. Consistent to the pattern observed in CD8⁺ T cells, a transient induction in CD44⁺ trained CD4⁺ T cells was detected following 4 weeks of *B. subtilis* natto NTU-18 administration, then a potential decrease of peripheral trained CD4⁺ T cells were observed after 10 weeks of treatment compare to PBS controls (Fig. 5a). In parallel, *B. subtilis* natto NTU-18-treated mice exhibited a potential decreased proportion of naïve CD4⁺ T cells and an increased frequency of effector memory CD4⁺ T cells at week 12 compared to the control group (Fig. 5b). Notably, *B. subtilis* natto NTU-18-treated atherosclerotic mice exhibited a sustained trend toward a higher proportion of regulatory CD25⁺CD4⁺ T cells throughout the course of atherosclerosis progression compared to PBS-treated controls (Fig. 5c). These findings suggest that daily *B. subtilis* natto NTU-18 administration promotes a shift toward a more activated CD4⁺ T cell phenotype, which may reflect an enhanced regulatory response, potentially contributing to immune modulation and stabilization of atherosclerotic lesions.

### Effects of *B.subtilis* natto NTU-18 administration on peripheral myeloid cell dynamics in atherosclerotic mice

**Fig. 6:**
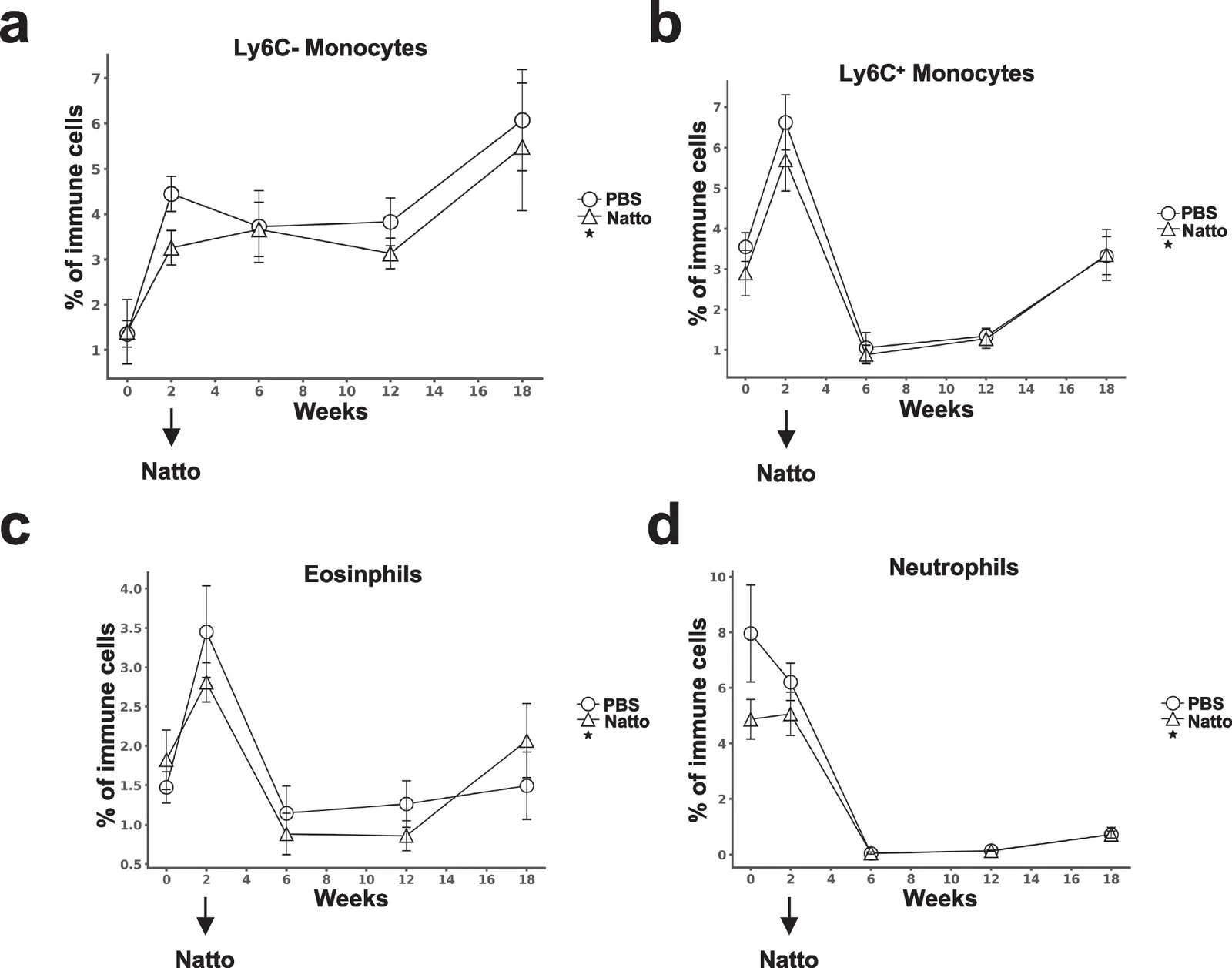
*B. subtilis* natto NTU-18 effects on peripheral myeloid cell populations during atherosclerosis progression. Representative density plots and longitudinal quantification of (**a**) Ly6C⁻ and (**b**) Ly6C⁺ monocytes, **c** eosinophils, and (**d**) neutrophils in peripheral blood at indicated time point. Data are presented as mean ± SEM (*n* = 7, PBS-treated group; *n* = 4, *B. subtilis* natto NTU-18-treated group)

The expansion of pro-inflammatory CD14⁺CD16⁺ monocytes, which are functionally analogous to Ly6C^hi^ monocytes in mice, has been closely linked to atherosclerosis progression (Hilgendorf et al. 2015). To investigate whether *B. subtilis* natto NTU-18 modulates peripheral myeloid cell populations, we analyzed circulating myeloid subsets in daily *B. subtilis* natto NTU-18-treated atherosclerotic mice compared to PBS-treated controls. No significant differences were observed in the frequencies of Ly6C⁻ or Ly6C⁺ monocyte subsets between the two groups (Fig. 6a and b). Similarly, the proportions of other myeloid populations, including eosinophils and neutrophils, remained unchanged following *B. subtilis* natto NTU-18 treatment (Fig. 6c and d). These findings suggest that the atheroprotective effects of *B. subtilis* natto NTU-18 are unlikely to be mediated through broad alterations in peripheral myeloid cell dynamics.

## Discussion

Probiotics are live microorganisms that confer health benefits to the host when administered in adequate amounts. *Lactobacillus plantarum* ATCC 14917 has been shown to attenuate atherosclerosis in *Apoe*^−/−^ mice by reducing proinflammatory cytokines such as TNF-α and IL-1β, and by alleviating oxidative stress via increased superoxide dismutase (SOD) activity (Hassan et al. 2020). Similarly, *Lactobacillus rhamnosus* GG mitigates atherosclerosis in high-fat diet-fed mice by modulating gut microbiota and enhancing ketone body synthesis, contributing to endothelial protection and metabolic regulation (Zhai et al. 2022). These findings suggest that daily probiotic supplementation may alleviate atherosclerosis progression through microbiota-mediated immune and metabolic modulation. However, the specific immune cell subsets involved in these protective effects remain poorly defined.

Our study provides new insights into the immunomodulatory mechanisms by which *B. subtilis* natto NTU-18 exerts protective effects in a murine model of atherosclerosis induced by AAV-mPCSK9-AAV and Western diet. One of the main bioactive components, vitamin K2, has been shown to exert anti-inflammatory effects on peritoneal macrophages from atherosclerotic mice (Kawamata et al. 2023). Surfactin, a biosurfactant produced by *Bacillus*, also attenuates atherosclerotic lesions in *Apoe*^⁻/⁻^ mice by reducing systemic and vascular inflammation, marked by decreased TNF-α, increased IL-10, and induction of CD4⁺CD25⁺Foxp3⁺ regulatory T cells (Gan et al. 2016). Although *B. subtilis* natto NTU-18 treatment did not significantly alter body weight or serum cholesterol levels in our study, a significant reduction in aortic root lesion area and a lower body weight gains were observed. This decoupling of lesion suppression from lipid-lowering mirrors findings from previous studies demonstrating anti-atherosclerotic effects of *B. subtilis* natto or its derivatives through non-lipid metabolic pathways, including enhanced thrombolytic activity and endothelial protection (Chen et al. 2022; Kawamata et al. 2023). Notably, *L. plantarum* ATCC 14917 also did not alter the serum lipid profile but was still effective in attenuating atherosclerosis progression (Hassan et al. 2020).

Natto consumption has been shown to modulate gut microbiota composition with downstream effects on host immunity. In atherosclerotic mice, natto administration enhanced microbial diversity, increased the abundance of *B. subtilis* natto in the cecum, and significantly reduced serum CCL2 levels, collectively contributing to reduced aortic plaque progression (Kawamata et al. 2023). A large-scale Japanese microbiota study further demonstrated that ingestion of *B. subtilis* variant natto, via either SONOMONO NATTO POWDER CAPSULES™ or traditional natto, significantly increased the relative abundance of beneficial genera such as *Bifidobacterium* and *Blautia*, particularly in individuals with low baseline levels (Kono et al. 2022). In addition, *B. subtilis* natto NTU-18 culture fluid was shown to inhibit *Enterococcus faecalis* biofilm formation by interfering with bacterial adherence, aggregation, exopolysaccharide synthesis, and cell envelope integrity, likely through transcriptional suppression of the WalK/WalR regulatory system and genes involved in envelope biosynthesis (Lin et al. 2021). These findings collectively highlight the multifaceted health benefits of *B. subtilis* natto, including its capacity to shape gut microbial ecology, modulate immune responses, and suppress pathogenic biofilms, underscoring its potential as a therapeutic probiotic in cardiovascular and infectious diseases.

Previous studies have shown that effector memory (EM) T cells contribute to atherosclerosis by recognizing lipid-associated antigens, such as oxidized LDL and apolipoprotein B100, thereby triggering localized inflammatory responses (Hermansson et al. 2010, 2011). These cells produce pro-inflammatory cytokines, migrate to atherosclerotic lesions via chemokine receptors such as CCR7, and expand under hypercholesterolemic conditions (Ammirati et al. 2012; Bromley et al. 2005). Their expansion correlates with increased LDL levels and is often accompanied by reduced regulatory T cell (Treg) function, further promoting plaque inflammation and instability. Notably, circulating EM T cells (effector memory T cells) have been associated with increased atherosclerosis and coronary artery disease in both humans and animal models, suggesting their pivotal role in atherogenesis (Ammirati et al. 2012). In this context, our study represents the most comprehensive analysis to date of circulating immune cell dynamics during *B. subtilis* natto NTU-18 intervention in a murine model of atherosclerosis. Using high-parameter flow cytometry to simultaneously assess 26 immune cell surface markers, we achieved an accurate and relatively unbiased characterization of peripheral immune cell phenotypes. This high-dimensional analysis revealed that *B. subtilis* natto NTU-18 induces transient yet dynamic immune remodeling. Specifically, we observed an early increase in peripheral myeloid cells and CD44⁺ trained CD8⁺ T cells at week 6, followed by a return to baseline by week 12. This pattern suggests a phase of acute immune activation, possibly reflecting antigen reprogramming or immune training, that transitions to a more homeostatic state (Figs. 3, 4). By week 12, we observed a significant enrichment of naïve and central memory CD8⁺ T cells alongside a reduction in effector memory subsets, indicating a shift toward a less inflammatory T cell landscape (Fig. 4). These findings are consistent with previous studies demonstrating that effector memory T cells perpetuate vascular inflammation, whereas naïve and central memory populations are linked to immune regulation and reduced atherosclerotic burden (Ammirati et al. 2012; Kyaw et al. 2017).

Similarly, peripheral CD4⁺ T cell subsets present a transient induction of trained phenotypes at week 6 and a progressive enrichment of regulatory CD25⁺CD4⁺ T cells through week 18 (Fig. 5). This sustained increase in Tregs aligns with previous work demonstrating their critical role in maintaining vascular homeostasis and suppressing pro-inflammatory immune activation (Ait-Oufella et al. 2006; Foks et al. 2015). The dual modulation of CD4⁺ and CD8⁺ T cells observed in our model suggests that *B. subtilis* natto NTU-18 exerts its immunomodulatory effects primarily through reprogramming of adaptive immune responses. Interestingly, while human CD14⁺CD16⁺ monocytes (equivalent to murine Ly6C^+^ monocytes) have been implicated in atherosclerotic plaque instability (Hilgendorf et al. 2015), our data showed no significant differences in Ly6C⁺ or Ly6C⁻ monocyte frequencies between groups. Likewise, neutrophils and eosinophils remained unchanged (Fig. 6). These findings suggest that the atheroprotective effects of *B. subtilis* natto NTU-18 are not mediated via broad suppression of myelopoiesis or innate immune cell trafficking, but rather through targeted modulation of T cell phenotypes.

In conclusion, our findings demonstrate that *B. subtilis* natto NTU-18 mitigates atherosclerosis through transient remodeling and long-term rebalancing of peripheral T cell responses, rather than through direct effects on lipid metabolism or broad myeloid cell suppression**.** While the study was conducted with a relatively small and uneven group size (PBS, *n* = 7; *B. subtilis* natto NTU-18, *n* = 4) due to technical limitations in repeated longitudinal sampling, the protective effects we observed were consistent across individuals and statistically significant. Notably, the reduced aortic plaque progression observed in our study is consistent with a previous study on certain strains of *B. subtilis* natto consumption (Kawamata et al. 2023). Taken together, our results provide mechanistic evidence supporting *B.* subtilis natto NTU-18 as a probiotic-based immunomodulatory intervention in atherosclerosis, and highlight its translational potential as a complementary strategy in cardiovascular disease management. Future studies with larger cohorts will be valuable to further validate and extend these findings.

## Data Availability

All data generated or analyzed during this study are included in this published article.
